# Systemic immune-inflammation index, stress hyperglycemia ratio and triglyceride-glucose index in acute ischemic stroke: an integrated inflammation–stress–metabolism perspective

**DOI:** 10.3389/fneur.2026.1911042

**Published:** 2026-08-14

**Authors:** Xin Lin, Baoai Wang

**Affiliations:** 1 Graduate School of Shanxi Medical University, Taiyuan, Shanxi, China; 2 Department of Neurology, Fenyang Hospital of Shanxi Province, Fenyang, Shanxi, China

**Keywords:** acute ischemic stroke, early neurological deterioration, insulin resistance, neuroinflammation, prognosis, stress hyperglycemia ratio, systemic immune-inflammation index, triglyceride-glucose index

## Abstract

**Background:**

Acute ischemic stroke (AIS) remains a major cause of mortality and long-term disability worldwide. Increasing evidence suggests that secondary brain injury after AIS is not driven by a single pathological process but results from the complex interaction among neuroinflammation, acute metabolic stress, and chronic metabolic dysfunction. The systemic immune-inflammation index (SII), stress hyperglycemia ratio (SHR), and triglyceride-glucose (TyG) index have emerged as readily available biomarkers reflecting these distinct yet interconnected pathophysiological pathways.

**Objective:**

To review the current evidence regarding the associations of SII, SHR, and TyG with disease severity, functional outcomes, and stroke-related complications in AIS, and to explore their complementary value within an integrated inflammation–stress–metabolism framework.

**Methods:**

This narrative review summarized relevant clinical and mechanistic studies investigating SII, SHR, and TyG in patients with AIS. Evidence was identified primarily through PubMed and supplemented by manual screening of reference lists.

**Results:**

Current evidence indicates that elevated SII is closely associated with inflammatory activation, stroke severity, poor functional outcomes, mortality, and various stroke-related complications. SHR reflects relative hyperglycemia induced by neuroendocrine stress and is associated with early neurological deterioration, hemorrhagic complications, cerebral edema, and adverse prognosis. As a surrogate marker of insulin resistance, TyG is associated with stroke severity, poor neurological recovery, recurrent stroke, and chronic vascular injury. Although these biomarkers originate from different biological pathways, inflammation, stress hyperglycemia, and insulin resistance interact extensively throughout the progression of AIS. Consequently, SII, SHR, and TyG provide complementary information regarding inflammatory burden, acute metabolic stress, and chronic metabolic vulnerability.

**Conclusion:**

SII, SHR, and TyG represent three interrelated yet distinct dimensions of AIS pathophysiology. Integrating these biomarkers may provide a more comprehensive assessment of patient risk than any single indicator alone and may improve risk stratification and prognostic evaluation. Future prospective studies are warranted to validate their combined predictive value and establish integrated risk assessment models for AIS.

## 1 Introduction

Stroke has consistently ranked prominently in the global burden of disease. According to data from the Global Burden of Disease Study, stroke is the second leading cause of death and the third leading cause of death and disability combined worldwide, with ischemic stroke accounting for approximately 65% of all new stroke cases (1). With the aging of the population and the growing number of stroke survivors, the burden of stroke-related neurological disability continues to increase. Consequently, the early identification of patients at high risk of neurological deterioration, complications, and poor functional recovery during the acute phase has become a major focus of research in acute ischemic stroke (AIS).

At present, intravenous thrombolysis and endovascular therapy remain the cornerstone treatments for AIS in the acute phase. However, their clinical benefits are constrained by several factors, including the therapeutic time window, eligibility criteria, and the risk of hemorrhagic transformation. Consequently, a substantial proportion of patients may still experience poor neurological recovery, stroke recurrence, or even death despite receiving guideline-recommended treatment (2, 3).

The NIHSS scale is widely used in clinical practice to assess the extent of neurological deficits in patients with acute ischemic stroke (AIS), but it essentially reflects the patient’s clinical neurological status. Because this scale relies in part on the physician’s subjective judgment, it has significant limitations when used to fully elucidate the complex inflammatory responses and metabolic disturbances that occur following a stroke. Given these limitations, identifying a simple, cost-effective, and reproducible peripheral blood biomarker is of great importance for early risk stratification and prognostic assessment in acute ischemic stroke (AIS).

Post-ischemic brain injury is not caused solely by vascular occlusion. Following cerebral ischemia, various pathophysiological processes—including neuroinflammatory responses, oxidative stress, disruption of the blood–brain barrier, and energy metabolism disorders—interact with one another and collectively contribute to secondary brain injury (4, 5). Immune-inflammatory dysregulation is closely associated with the pathophysiological process of AIS, stroke severity, functional outcomes, and complications (5). At the same time, insulin resistance and disturbances in glucose and lipid metabolism can also affect clinical outcomes in patients with AIS (6, 7).

Among inflammatory markers, the systemic immune-inflammation index (SII), which integrates information on platelets, neutrophils, and lymphocytes, has drawn attention for its ability to simultaneously reflect inflammation activation, immune imbalance, and thrombosis. Inflammatory responses are a key mechanism underlying brain injury in ischemic stroke, and associated inflammatory markers are linked to adverse outcomes such as functional disability, death, recurrence, and cardiovascular events (5). Previous studies have shown that elevated SII levels are closely associated with stroke risk, the severity of neurological deficits, and poor outcomes (4). This suggests that the composite index has potential utility in assessing the severity of AIS and predicting prognosis. At the same time, following cerebral ischemia, the body may experience stress-induced hyperglycemia due to activation of the neuroendocrine stress response; a persistent state of hyperglycemia can further exacerbate oxidative stress and inflammatory responses, thereby promoting the development of secondary brain injury (8–10). The stress-induced hyperglycemia ratio (SHR) partially accounts for baseline glycemic status and may therefore better reflect relative hyperglycemia in acute disease states (8, 9). In addition, insulin resistance and chronic glucose-lipid metabolism abnormalities are closely associated with atherosclerosis, endothelial dysfunction, and inflammatory responses. The triglyceride-glucose index (TyG), as a surrogate marker of insulin resistance, has also been shown in recent years to be associated with poor outcomes in AIS (6, 7, 11).

Although SII, SHR, and TyG have all been shown to be associated with the severity and prognosis of AIS, most existing studies have focused on a single indicator, and there is a lack of systematic analysis of the interrelationships among these three measures and their complementary value. Since AIS is not driven by a single pathway but rather results from the overlapping effects of inflammatory, stress, and metabolic processes, a single biomarker may not fully account for its pathological heterogeneity. This article reviews the research evidence on SII, SHR, and TyG in AIS and discusses their potential complementary value from the perspective of an integrated inflammation-stress-metabolism framework.

## 2 Literature identification and selection

This review was conducted as a narrative review to summarize the current evidence regarding the clinical significance of the systemic immune-inflammation index (SII), stress hyperglycemia ratio (SHR), and triglyceride-glucose (TyG) index in acute ischemic stroke (AIS). Relevant literature published up to May 2026 was identified primarily through PubMed.

The search strategy combined Medical Subject Headings (MeSH) and free-text terms, including “acute ischemic stroke,” “systemic immune-inflammation index,” “SII,” “stress hyperglycemia ratio,” “SHR,” “triglyceride-glucose index,” “TyG,” “neuroinflammation,” “insulin resistance,” “early neurological deterioration,” “functional outcome,” and “prognosis.”

Original clinical studies, systematic reviews, and meta-analyses investigating the associations of these biomarkers with stroke severity, functional outcomes, mortality, or stroke-related complications were preferentially included. Conference abstracts without full-text availability, duplicate publications, case reports, editorials, and studies unrelated to AIS were excluded. The reference lists of relevant articles were also manually screened to identify additional eligible studies that were not captured during the initial search.

Particular attention was paid to recently published high-quality cohort studies, systematic reviews, and meta-analyses to ensure that the evidence presented reflects the latest advances in this rapidly evolving field.

## 3 The SII and acute ischemic stroke

### 3.1 The pathophysiological basis of the SII

Secondary brain injury following AIS is closely associated with a neuroinflammatory response. Following cerebral ischemia, necrotic tissue releases large amounts of damage-associated molecular patterns, which activate microglia and the peripheral immune system, thereby inducing inflammatory cascades, oxidative stress, and disruption of the blood–brain barrier (12). Therefore, neuroinflammation is not only involved in the process of acute brain injury but is also closely associated with the progression of infarction, reperfusion injury, and long-term neurological recovery (13).

Neutrophils are among the first peripheral inflammatory cells to be recruited to ischemic tissue. They further amplify the inflammatory response by releasing reactive oxygen species, proteases, and neutrophil extracellular traps. Previous studies have shown that neutrophil-derived matrix metalloproteinase-9 can degrade collagen IV and disrupt the blood–brain barrier, thereby promoting vasogenic edema and secondary brain injury (14). At the same time, T lymphocytes are involved in the process of secondary neuroinflammation following a stroke; experimental studies have shown that T-cell deficiency can reduce platelet adhesion within cerebral microvessels and decrease infarct volume (15). In addition to participating in thrombus formation, platelets can also interact with neutrophils and monocytes, promoting the amplification of inflammation and microcirculatory dysfunction (13). Increased blood–brain barrier permeability is independently associated with poor functional outcomes in patients with acute ischemic stroke (16).

Based on the above biological background, Hu et al. initially proposed the use of SII to predict postoperative outcomes in patients with hepatocellular carcinoma (17). Because the SII combines platelet, neutrophil, and lymphocyte counts, it reflects inflammatory activation, immunosuppression, and a prothrombotic state in a single index. In AIS, this composite measure may provide broader information than a single inflammatory marker and improve the ability to predict clinically relevant outcomes (18).

### 3.2 Severity and functional prognosis of SII and AIS

An increase in SII is significantly associated with an increase in AIS severity (19–22); numerous studies consistently support this association. Huang et al. analyzed 234 patients with acute ischemic stroke and found that SII levels were significantly elevated in the moderate-to-severe stroke group and were positively correlated with NIHSS scores (19). Subsequent studies and meta-analyses have further supported the consistent association between SII and AIS severity (20–22). Compared to the severity of AIS, there is currently a greater body of research evidence regarding SII and functional prognosis. High SII is associated with short- and long-term adverse functional outcomes in patients undergoing intravenous thrombolysis, mechanical thrombectomy, and conventional treatment for acute ischemic stroke (20, 23–25). Among patients who underwent intravenous thrombolysis, Ma et al. found that, after adjusting for age, NIHSS score, and comorbidities, a high SII remained an independent risk factor for adverse outcomes at 90 days (20). Subsequent studies have further confirmed that the SII may be superior to traditional inflammatory ratios such as the NLR, PLR, and LMR in predicting poor outcomes following thrombolysis (23, 24). In patients undergoing mechanical thrombectomy, the SII also demonstrates high predictive value, adding that the SII further improves the performance of clinical prediction models (25).

In addition to single-point measurements, recent studies have begun to focus on the clinical significance of dynamic changes in SII. Through cluster analysis, Huang et al. found that marked fluctuations in SII within 7 days following AIS also indicate a poor long-term prognosis, suggesting that persistent inflammatory imbalance may affect neurological recovery (26). Existing data also link high SII to an increased risk of death. An analysis by Wu et al. based on the MIMIC-IV database found that patients in the top quartile of SII had a significantly increased risk of death at both 30 and 90 days (27). Subsequent studies further confirmed that high SII is significantly associated with the risk of all-cause mortality at 90 days and 1 year (21).

### 3.3 Complications and secondary injuries associated with SII and AIS

In addition to stroke severity and functional prognosis, the SII is also closely associated with various complications of acute ischemic stroke (AIS) and the process of secondary damage. The inflammatory response following a stroke can lead to immune dysregulation, microcirculatory dysfunction, and systemic organ involvement. Therefore, elevated SII levels may indicate an increased risk of secondary damage and complications (12, 13).

Stroke-associated pneumonia (SAP) is one of the most common and most serious complications of acute ischemic stroke (AIS). Post-stroke immunosuppression and systemic inflammation activation jointly participate in the occurrence process of SAP, while SII can simultaneously reflect the increase of neutrophils, the decrease of lymphocytes, and the activation of platelets (12, 28, 29). Multiple studies have found that elevated SII levels are significantly associated with an increased risk of SAP, and that there is a dose–response relationship (28, 29). In addition, combining SII with the traditional clinical score A2DS2 can further improve the predictive performance of SAP (28). Furthermore, the effects of inflammatory activation may not be limited to the infection itself. In patients with AIS complicated by pulmonary infection, SII was positively correlated with the mRS score at 6 months post-onset, suggesting that it may be associated with subsequent neurological recovery (30). Systemic inflammation may mediate the association between stress-induced hyperglycemia and SAP, as well as long-term poor outcomes (31).

The activation of the inflammatory response following AIS can further contribute to the disruption of the blood–brain barrier, microcirculatory dysfunction, and reperfusion injury (32–34). High SII levels are significantly associated with an increased risk of early neurological deterioration (END) and hemorrhagic transformation (HT) (22). In patients undergoing reperfusion therapy, SII has also demonstrated predictive value for reperfusion failure and poor postoperative functional outcomes (25, 35). However, SII still has certain limitations in predicting pathological processes associated with local mass effects, such as malignant cerebral edema (24), suggesting that its predictive value for different types of secondary brain injuries may vary.

The effects of inflammatory activation extend beyond the acute phase of AIS. Elevated SII levels have been associated with both acute complications and long-term outcomes, including stroke-heart syndrome (SHS), post-stroke cognitive impairment (PSCI), stroke recurrence, and long-term mortality (18, 21, 27, 36). Patients with PSCI consistently exhibit higher baseline SII levels, which correlate with the severity of cognitive impairment (36). Chronic neuroinflammation directly affects synaptic plasticity, neurotransmitter metabolism, and brain network reorganization, providing a potential mechanistic explanation for the association between SII and delayed neurological sequelae. Furthermore, systemic inflammatory responses after AIS contribute to cardiovascular complications, and elevated admission SII has been independently associated with an increased risk of SHS within 14 days after stroke (18). Collectively, these findings indicate that SII is associated with both acute and long-term neurological and systemic outcomes in patients with AIS.

## 4 SHR and acute ischemic stroke

### 4.1 Pathophysiological basis of SHR

Stress-induced hyperglycemia is one of the common metabolic abnormalities following an acute stroke. Following cerebral ischemia, the body activates the hypothalamic–pituitary–adrenal (HPA) axis and the sympathetic nervous system, triggering a massive release of catecholamines and glucocorticoids, which in turn promote hepatic glucose output, exacerbate peripheral insulin resistance, and elevate blood glucose levels (9). Persistent hyperglycemia can further induce oxidative stress, disruption of the blood–brain barrier, lactate accumulation, and inflammatory cascades, thereby exacerbating ischemic penumbra damage and reperfusion injury (8, 10).

Traditionally, stress-induced hyperglycemia has been assessed primarily based on absolute glucose levels; however, patients with different baseline glucose levels have varying tolerances to the same glucose level. Snarska et al. found that the blood glucose threshold for predicting the risk of stroke-related death was significantly lower in non-diabetic patients than in diabetic patients, suggesting that absolute blood glucose levels alone do not reflect the true stress response (37). To address this limitation, Roberts et al. proposed the SHR in 2015; this index is typically calculated by dividing the blood glucose level at admission by the average blood glucose level estimated from HbA1c. Compared with traditional blood glucose measures, the SHR better reflects relative hyperglycemia in acute disease states (38). By incorporating chronic glycemic status reflected by HbA1c, SHR enables a more individualized assessment of stress-induced hyperglycemia than admission blood glucose alone. This enables SHR to distinguish acute stress hyperglycemia from chronically elevated glucose levels, thereby reducing the confounding effects of pre-existing diabetes and long-term glycemic control. Consistent with this concept, recent studies have shown that SHR outperforms admission blood glucose and other glucose-related metrics in predicting functional outcomes and complications after AIS, supporting its value as a more robust indicator of acute metabolic stress (39).

In recent years, a growing body of research has found that SHR is closely associated with outcomes such as functional prognosis, mortality, hemorrhagic transformation, cerebral edema, and early neurological deterioration in patients with AIS. Song et al. further noted in their systematic review that there may be a J-shaped association between SHR and the risk of death from AIS, with most risk thresholds concentrated between 0.8 and 1.2, suggesting that a moderately stable glucose metabolism may be more conducive to brain tissue recovery (40).

In addition, there is a close association between stress-induced hyperglycemia and the inflammatory response. Elevated SHR is significantly associated with neutrophil count, NLR, and SIRI (41). Inflammatory markers may partially mediate the association between SHR and poor outcomes in AIS (42); this suggests that the inflammation-metabolism coupling may be one of the key mechanisms underlying the impact of SHR on stroke outcomes.

### 4.2 Severity and functional prognosis of SHR and AIS

Unlike the SII, which primarily reflects the inflammatory burden, the SHR focuses more on the relative increase in blood glucose levels under acute stress conditions. Elevated SHR values are often associated with more severe neurological impairment in patients with AIS. Patients with elevated SHR scores tend to have higher NIHSS scores, a higher incidence of impaired consciousness, and an increased risk of Early Neurological Deterioration (END) (43, 44). Among patients receiving intravenous thrombolysis, Wang et al. were the first to report that the risk of END was significantly higher in the high SHR group, and this finding was even more pronounced in patients with diabetes (45). Inflammation may be involved in SHR-related secondary brain injury. Yang et al. found that CRP level and white blood cell count could partially mediate the relationship between SHR and END through mediation analysis (46). These findings suggest that inflammatory responses may contribute to secondary brain damage caused by stress-induced hyperglycemia. However, the relationship between SHR and END is not entirely consistent across different studies. Some studies, after excluding patients with hemorrhagic transformation and cardioembolism, have found that a lower SHR is actually associated with an increased risk of END (47). This inconsistency suggests that stroke subtypes, reperfusion status, patient selection, and the definition of END may all influence the prognostic significance of SHR. In addition, SHR is associated with severe impaired consciousness and long-term mortality risk. Its predictive performance may be superior to that of traditional admission blood glucose measures (48). Overall, the available evidence supports SHR as a marker of acute metabolic stress that is closely associated with neurological severity and the risk of END in AIS.

A growing body of evidence indicates that elevated SHR is associated with adverse short- and long-term outcomes in patients with AIS. A high SHR is significantly associated with increased short- and long-term adverse functional outcomes, higher mortality rates, and poorer neurological recovery (8, 40, 43, 44). Among patients in general AIS and ICU settings, those with high SHR had a significantly increased risk of death at both 30 and 90 days (49, 50). Some studies suggest that this association may be more pronounced in non-diabetic individuals (49). In patients receiving reperfusion therapy, SHR also demonstrates high predictive value. Previous studies have found that SHR is a better predictor of outcomes in patients with acute ischemic stroke (AIS) undergoing thrombolysis than traditional glycemic markers (51). In patients undergoing mechanical thrombectomy (MT) and endovascular treatment (EVT), a high SHR is associated with an increased risk of functional dependence, mortality, and symptomatic intracranial hemorrhage (44, 52). It has also been reported to be associated with a decrease in recanalization rates and reperfusion failure (44). It is worth noting that the relationship between SHR and AIS prognosis may not follow a simple linear pattern. A dose–response meta-analysis revealed a J-shaped relationship between SHR and the risk of poor prognosis and mortality, with a significant increase in risk when SHR exceeds approximately 1.0 (53). Regarding the impact of diabetic status on the predictive performance of SHR, current research findings are not entirely consistent (54). Nevertheless, compared with admission glucose alone, SHR can partially account for differences in baseline glycemic status and may therefore better reflect the actual metabolic stress burden after stroke. These characteristics may explain its superior prognostic performance across different AIS populations and treatment settings.

### 4.3 SHR and AIS-related complications and secondary injury

Stress-induced hyperglycemia can exacerbate reperfusion injury following acute ischemic stroke (AIS) by promoting oxidative stress, disrupting the blood–brain barrier, and causing endothelial dysfunction (9, 10).

Elevated SHR is associated with an increased risk of HT, sICH, cerebral edema, and poor functional outcomes (8, 44, 54–56). Some studies have found that elevated SHR is significantly associated with an increased risk of HT (54, 55), and reperfusion therapy cohorts have also shown an association with symptomatic intracranial hemorrhage (8, 44). Higher SHR is also associated with increased severity of cerebral edema and an elevated risk of moderate-to-severe cerebral edema (56). These findings suggest that SHR may contribute to secondary brain injury in AIS through mechanisms related to blood–brain barrier disruption, microcirculatory dysfunction, and reperfusion injury.

Beyond its association with secondary brain injury, SHR may provide greater prognostic value when combined with inflammatory markers, clinical scores, or other metabolic indicators. Several studies have shown that incorporating SHR together with inflammatory markers (e.g., SII or SIRI) or established clinical scoring systems further improves the prediction of adverse outcomes and stroke-associated complications after AIS (31, 57). Importantly, the prognostic significance of SHR may not be solely attributable to hyperglycemia itself. Patients with elevated SHR levels often exhibit a more pronounced inflammatory response, and inflammatory markers have been reported to partially mediate the association between SHR and poor outcomes as well as END (41, 42, 46). These findings suggest that SHR may reflect inflammation–metabolism coupling rather than isolated glucose abnormalities. As a marker of relative hyperglycemia, SHR accounts for baseline glycemic status and may therefore better capture the actual metabolic stress burden after stroke than admission glucose alone. Current evidence links elevated SHR to stroke severity, END, hemorrhagic complications, cerebral edema, and mortality, highlighting its potential utility in risk stratification. Furthermore, SHR may provide additional prognostic information when interpreted together with inflammatory and metabolic markers, suggesting that its clinical significance extends beyond the assessment of hyperglycemia alone. Nevertheless, several limitations remain. The calculation of SHR has not yet been fully standardized, different studies have used varying glucose measurements and HbA1c conversion methods, and the influence of diabetic status remains controversial. In addition, most available evidence is derived from observational studies, making causal inference difficult. Future prospective studies should establish standardized calculation methods, determine optimal risk thresholds, and further evaluate the added value of integrating SHR with inflammatory and metabolic biomarkers.

## 5 TyG and acute ischemic stroke

### 5.1 Pathophysiological basis of TyG

Insulin resistance (IR) is a key pathological basis for the onset and progression of atherosclerosis and cerebrovascular diseases. A prolonged state of IR can promote endothelial dysfunction, oxidative stress, chronic low-grade inflammation, and dyslipidemia, thereby accelerating the development of atherosclerosis and increasing the risk of thrombosis. In recent years, triglyceride-glucose index (TyG) has been widely used as a surrogate marker for IR due to its simplicity of calculation and relative stability in routine clinical practice (7).

In patients with AIS, IR not only contributes to the stroke process but may also affect post-ischemic microcirculatory perfusion, blood–brain barrier (BBB) integrity, and reperfusion injury (7). Based on a CT perfusion study, Wang et al. found that the TyG index is significantly associated with blood–brain barrier (BBB) permeability, and that BBB disruption fully mediates the relationship between TyG and poor 90-day outcomes (58). This finding suggests that BBB disruption may be an important pathway linking IR to adverse outcomes after AIS.

Furthermore, there is a close interaction between IR and the inflammatory response. Huang et al. found that the neutrophil-to-platelet ratio (NPR) partially mediates the association between TyG and poor prognosis; although SII did not reach statistical significance as a mediator, it showed an upward trend in patients with high TyG levels, suggesting that metabolic dysfunction and inflammatory activation may jointly contribute to the AIS injury process through distinct yet interrelated mechanisms (59).

Unlike SHR, which reflects acute metabolic stress, TyG is closer to long-term metabolic abnormalities and chronic vascular injury. Therefore, the two may focus on the baseline vascular vulnerability and acute secondary injury process, respectively (7, 9).

### 5.2 TyG and AIS severity and early neurological deterioration

Compared with SII and SHR, TyG primarily reflects chronic insulin resistance. Higher TyG is associated with more severe neurological deficits and a higher risk of END in AIS (60–62). Liu et al. observed an independent linear dose–response relationship between TyG and END in patients with acute mild AIS (60). In patients undergoing intravenous thrombolysis, high TyG was also associated with a reduced probability of early neurological improvement (ENI) (61).

However, some studies have shown that when TyG is used alone for risk prediction, its overall discriminative ability is still relatively limited (63, 64). This suggests that simple metabolic indicators are difficult to fully reflect the complex pathological process after AIS, and combined with other inflammatory or stress indicators may be more valuable. There is now substantial evidence regarding the prognostic significance of TyG and AIS scores. Most studies indicate that a high TyG score is significantly associated with poor short- and long-term functional outcomes, an increased risk of death, and poor neurological recovery (7, 59, 65–69). Among patients with AIS undergoing reperfusion therapy, elevated TyG has been associated with poor 90-day functional outcomes, an increased risk of death, early neurological deterioration (END), and poorer neurological recovery (59, 65, 66, 69). These associations appear to be more pronounced in cohorts undergoing endovascular treatment and mechanical thrombectomy (59, 66).

It is worth noting that there may be a nonlinear relationship between TyG and the prognosis of AIS. Some studies have found that once TyG exceeds a certain threshold, the trend of increased risk gradually levels off, suggesting the possible existence of a “ceiling effect” (67). The prognostic value of TyG may also vary with age, diabetes status, and stroke subtype, showing a stronger association in younger and non-diabetic patients (68). Overall, current evidence supports TyG as an important metabolic prognostic indicator for AIS; however, its predictive power remains limited when used in isolation and is better suited as part of a multidimensional risk assessment system.

### 5.3 TyG and AIS-related complications and secondary injury

IR may exacerbate BBB disruption and reperfusion injury following acute ischemic stroke (AIS) by promoting oxidative stress, endothelial dysfunction, and inflammation (7). CT perfusion studies have shown that TyG is significantly positively correlated with BBB permeability in the core infarct zone, suggesting that BBB disruption may mediate the association between TyG and adverse outcomes (58). In patients receiving reperfusion therapy, high TyG is associated with an increased risk of END (61, 70). In contrast, the association between TyG and symptomatic intracranial hemorrhage is less consistent than that with SHR. Some endovascular therapy studies have reported that high TyG increases the risk of hemorrhage, whereas several intravenous thrombolysis studies have found no significant association (59, 65). These inconsistent results suggest that TyG may reflect a chronic state of vascular fragility rather than an acute propensity for hemorrhage (7).

In addition to acute-phase injury, TyG is also associated with long-term vascular events and neuropsychiatric complications in AIS. In patients with non-diabetic small-vessel occlusion, elevated TyG is associated with an increased risk of recurrence (71). In addition, TyG has been independently associated with symptomatic intracranial atherosclerotic stenosis and vulnerable plaque formation, further suggesting that long-term metabolic abnormalities may contribute to the development and progression of intracranial atherosclerosis (72). Recent studies have also linked elevated TyG to post-stroke cognitive impairment and post-stroke depression (73, 74). Since chronic IR can affect cerebral vascular function, neuroinflammation, and neural network reorganization, elevated TyG levels may indicate a persistent metabolic-inflammatory imbalance following acute ischemic stroke (AIS).

Current research generally agrees that a single metabolic marker is insufficient to comprehensively assess the complex pathophysiological processes following AIS, and the importance of multidimensional combined assessment is increasingly being recognized. Existing evidence indicates that combining TyG with GAR, TG/HDL-C, or NIHSS significantly improves predictive performance (11, 42, 63, 70, 75). Overall, TyG is an important marker reflecting chronic metabolic vulnerability and vascular metabolic impairment in patients with acute ischemic stroke (AIS). Its advantages include ease of calculation, high accessibility, and a certain degree of biological plausibility as a surrogate marker for IR. TyG is associated with stroke severity, END, functional outcomes, stroke recurrence, atherosclerotic burden, and certain neuropsychiatric complications, suggesting that it is not only related to acute-phase clinical outcomes but may also influence long-term vascular risk. However, TyG is not a direct measurement of IR, and there remain certain discrepancies between it and HOMA-IR. Furthermore, its values may be influenced by fluctuations in triglyceride levels, lipid-lowering therapy, fasting status, and dietary factors, and its predictive performance may vary with age, diabetic status, and stroke mechanism. Some studies have found that the independent predictive power of TyG is somewhat diminished after adjusting for clinical severity and traditional vascular risk factors. Therefore, TyG is more suitable as a component of a comprehensive risk assessment system rather than as a standalone prognostic indicator.

## 6 An integrated inflammation–stress–metabolic model in AIS

Secondary brain injury following AIS is not driven by a single pathological process, but rather results from the combined effects of inflammatory responses, metabolic stress, and long-term metabolic abnormalities (6, 9, 12, 13). Existing studies indicate that although SII, SHR, and TyG originate from different biological backgrounds, the pathological processes reflected by these three models are closely interrelated (6, 7, 9). Inflammation can induce stress-induced hyperglycemia and insulin resistance by promoting cytokine release and disrupting insulin signaling pathways. Persistent hyperglycemia and insulin resistance, in turn, further exacerbate oxidative stress, vascular endothelial damage, and neuroinflammation, thereby creating a mutually reinforcing vicious cycle (6, 9, 10, 13). This bidirectional interaction supports an integrated inflammation-stress-metabolism perspective for understanding the pathophysiology of AIS and may facilitate a more comprehensive assessment of patient risk status.

**Figure 1:**
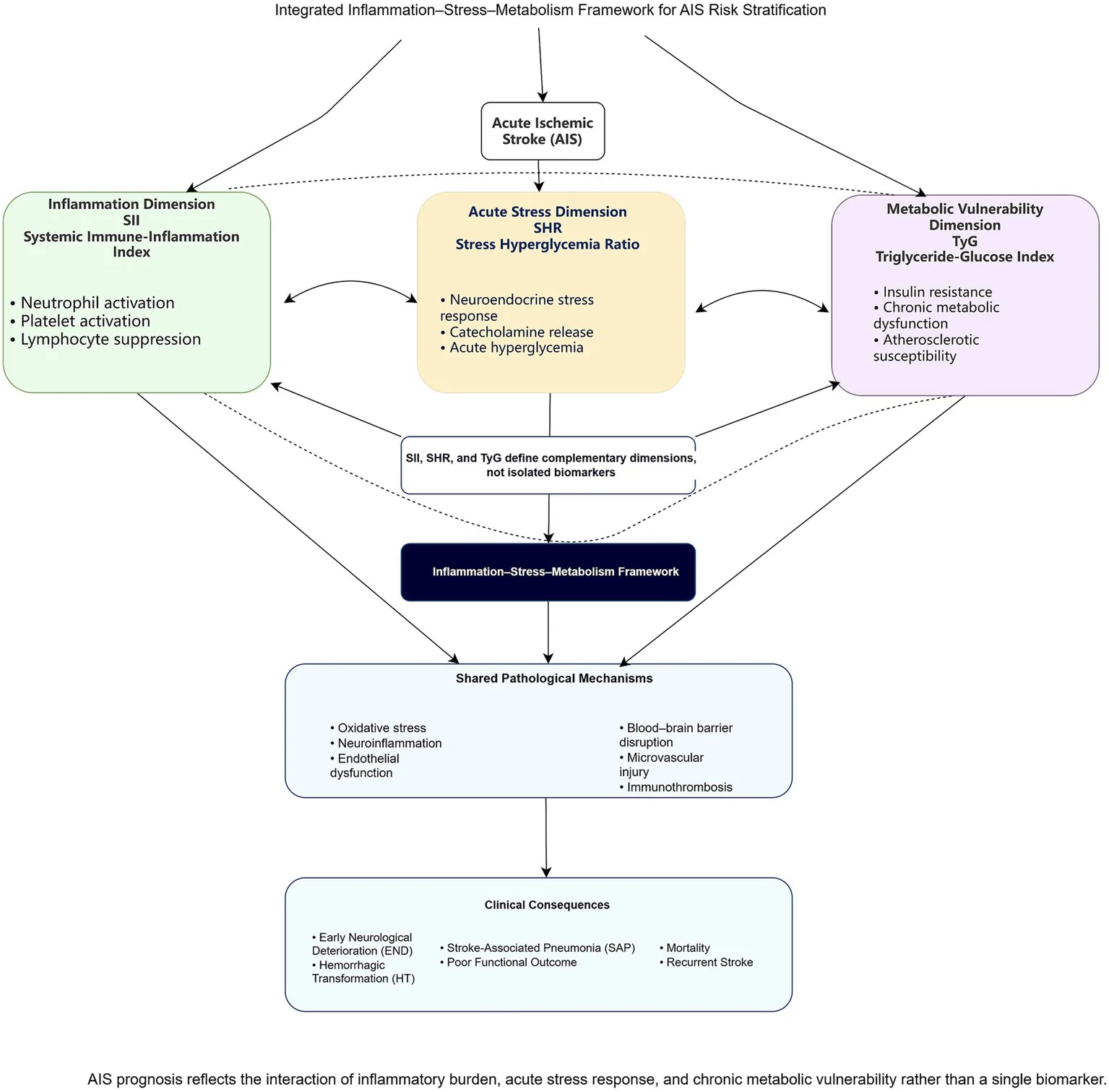
Integrated inflammation–stress–metabolism framework for risk stratification in acute ischemic stroke.

Within this framework, SII, SHR, and TyG should not be regarded as redundant biomarkers reflecting the same pathological process, but rather as complementary indicators representing distinct dimensions of AIS pathophysiology. SII primarily reflects post-stroke inflammatory activation, immune dysregulation, and thromboinflammatory responses. SHR reflects acute neuroendocrine stress and relative stress hyperglycemia following cerebral ischemia, whereas TyG is more closely associated with chronic insulin resistance, metabolic dysfunction, and pre-existing vascular vulnerability. Although these biomarkers originate from different biological pathways, they converge on several common downstream mechanisms, including blood–brain barrier disruption, oxidative stress, microvascular dysfunction, and secondary brain injury. Consequently, integrating SII, SHR, and TyG may provide a more comprehensive characterization of inflammatory burden, acute metabolic stress, and chronic metabolic vulnerability than any single biomarker alone. This complementary value may be particularly important in patients undergoing reperfusion therapies, especially endovascular thrombectomy (EVT). Despite successful recanalization, a substantial proportion of patients continue to experience unfavorable outcomes because of reperfusion injury, microvascular dysfunction, blood–brain barrier disruption, and persistent neuroinflammation. As SII, SHR, and TyG reflect inflammatory activation, acute metabolic stress, and chronic metabolic vulnerability, respectively, their combined assessment may provide prognostic information beyond procedural success alone and facilitate more individualized prognostic assessment and risk stratification after EVT. This multidimensional framework may help explain the marked clinical heterogeneity observed among AIS patients and improve the accuracy of risk stratification and prognostic assessment. Rather than searching for additional isolated biomarkers, future AIS risk assessment may benefit more from multidimensional models integrating inflammatory, stress-related, and metabolic pathways. In this context, SII, SHR, and TyG may represent the core components of an integrated inflammation–stress–metabolism framework capable of capturing the biological heterogeneity of AIS. The relationships among SII, SHR, TyG, and the integrated inflammation–stress–metabolism framework are summarized in Figure 1.

## 7 Current challenges and future perspectives

Despite the growing body of evidence supporting the clinical value of SII, SHR, and TyG in AIS, several important limitations should be acknowledged. First, considerable variability exists in biomarker measurement protocols across studies. Although most studies assessed biomarkers at hospital admission, variations in sampling time and assessment procedures may contribute to heterogeneity in the reported findings.

Second, substantial heterogeneity exists in the definitions of clinical outcomes. For example, poor functional outcome has been defined using different modified Rankin Scale (mRS) cutoffs, and early neurological deterioration (END) has been defined using different NIHSS criteria across studies. These inconsistencies complicate direct comparisons across studies and may partly explain the variability in the reported prognostic performance of these biomarkers.

Third, follow-up duration varied considerably across studies, ranging from in-hospital outcomes to 30-day, 90-day, and longer-term follow-up. Such differences may contribute to variability in the reported prognostic performance of these biomarkers. Furthermore, the current evidence is predominantly derived from retrospective observational studies, and many published cohorts originate from Asian populations, which may limit the generalizability of these findings to populations of different ethnic backgrounds and healthcare settings. Moreover, evidence from large multicenter prospective cohorts involving non-Asian populations remains limited. Additional validation studies across different ethnic groups and healthcare systems are needed before these biomarkers can be widely implemented in routine clinical practice.

Finally, the reported cutoff values for SII, SHR, and TyG vary considerably across studies. This variability is not unexpected, as the optimal thresholds are influenced by differences in study populations, clinical settings, outcome definitions, biomarker measurement timing, and statistical methods used for threshold determination. Accordingly, caution should be exercised when directly comparing or applying study-specific thresholds in clinical practice. Given the complexity of AIS pathophysiology, future studies should evaluate whether integrated prediction models that incorporate inflammatory, stress-related, and metabolic biomarkers and account for context-specific thresholds can further improve risk stratification and prognostic assessment.

## 8 Conclusion

SII, SHR, and TyG represent three distinct yet interconnected dimensions of AIS pathophysiology. SII primarily reflects inflammatory activation and immunothrombotic responses, SHR reflects acute metabolic stress and relative stress hyperglycemia, whereas TyG reflects chronic insulin resistance and long-term metabolic vulnerability. Although derived from different biological pathways, these biomarkers are linked through complex interactions among inflammation, stress responses, and metabolic dysfunction, and collectively contribute to secondary brain injury and adverse clinical outcomes after AIS.

Current evidence suggests that SII, SHR, and TyG are each associated with stroke severity, early neurological deterioration, functional outcomes, and a variety of stroke-related complications. More importantly, their complementary biological characteristics support an integrated inflammation–stress–metabolism framework, which may provide a more comprehensive assessment of patient risk than any single biomarker alone. Nevertheless, existing evidence remains limited by study heterogeneity, inconsistent cutoff values, and a lack of large-scale prospective validation. Future studies should further evaluate the incremental value of combining these biomarkers with clinical and imaging indicators and establish standardized multidimensional prediction models to facilitate more precise risk stratification and individualized management of AIS. Future multicenter studies involving diverse ethnic populations are also warranted to improve the external validity and clinical applicability of these biomarkers.
